# A Review of Temporal Self-Perceptions Among Emerging Adults: The Significance of Demographics and a Global Crisis on Psychological and Achievement Benefits

**DOI:** 10.3390/bs15040471

**Published:** 2025-04-05

**Authors:** Samantha L. McMichael, Virginia S. Y. Kwan

**Affiliations:** Department of Psychology, Arizona State University, Tempe, AZ 85287, USA

**Keywords:** intertemporal decision-making, emerging adults, longitudinal outcomes, psychological well-being, academic success

## Abstract

In industrialized societies, emerging adulthood is a unique developmental stage between adolescence and adulthood (i.e., 18 to 29 years old), where individuals continuously encounter decisions that have important consequences that unfold over time (i.e., intertemporal decisions). The present review paper had three aims. The first aim was to provide a brief overview of emerging adulthood as a developmental stage and present a rationale for the importance of understanding the relationship between temporal self-perceptions and longitudinal outcomes in emerging adults. The second aim was to review evidence for a proposed model demonstrating the connection between three domains of temporal self-perceptions—future self-perceptions, longitudinal changes in future self-perceptions, and continuity between temporal selves (i.e., past-to-future)—, self-regulatory processes, and positive downstream consequences (e.g., psychological well-being and academic success) in emerging adults. Specifically, this targeted review sought to highlight research exploring the longitudinal processes in these relationships (e.g., changes in temporal self-perceptions over time and the relationship between temporal self-perceptions and longitudinal outcomes) and testing how these relationships function amidst a large-scale challenge (i.e., the COVID-19 pandemic). Given the heterogeneity of emerging adults as a demographic group, the third aim was to review research exploring demographic factors (e.g., gender and socioeconomic status) as a potential moderator of these relationships. The review provided support for the proposed model, highlighting the importance of perceptions of temporal selves in predicting a range of positive long-term outcomes, including self-regulatory processes, psychological well-being, and achievement. Additionally, the review demonstrated preliminary support for the significance of crucial demographic factors (e.g., gender and socioeconomic status) in understanding the nature of these relationships in emerging adults. Finally, the review suggests future directions to extend this growing literature and broaden the understanding of these relationships.

## 1. Overview

In industrialized societies, where individuals often have the benefit of economic and social environments that allow for delays in taking on the full responsibilities of adulthood, emerging adulthood (i.e., approximately 18 to 29 year olds) is the developmental stage between the restrictions of adolescence and adult obligations that encompasses changes in identity and personal exploration (Arnett, 2000, 2004; Syed & Mitchell, 2013). Entering adulthood, individuals in this stage (i.e., emerging adults) are often focused on the potential for their future endeavors and perceive their future as relatively long-term when compared to older adults (Carstensen et al., 2020; Demiray & Bluck, 2014). Emerging adults continuously encounter decisions that have important consequences that unfold over the course of time (i.e., intertemporal decisions). While it may be optimal for emerging adults to focus on their future when making intertemporal decisions, these individuals often face both routine (e.g., receiving a poor grade) and large-scale (e.g., a global pandemic; recessions) challenges that complicate their decision-making processes. These intertemporal decisions are crucial as their consequences may play, out not just over time, but over the course of their lifetime. The consequences of these decisions may have a large impact on emerging adults’ psychological well-being, mental health, and success in the long term.

Temporal self-perceptions—individuals’ perceptions of themselves across points in time (e.g., past, present, and future)—are an important predictor of self-regulation processes and, ultimately, of how individuals approach making intertemporal decisions and persist through present difficulties (e.g., financial decisions to save for the future and procrastination; Bartels & Rips, 2010; Blouin-Hudon & Pychyl, 2015; Ersner-Hershfield et al., 2009a, 2009b). Given the frequency of intertemporal decision-making and challenges in emerging adults’ lives, it may be particularly crucial to explore the relationship between temporal self-perceptions and long-term outcomes for individuals in this developmental stage.

A large part of the recent research on temporal self-perceptions focuses on the relationship between the present and future self and the implications of that relationship (e.g., Burrow et al., 2020; Ersner-Hershfield et al., 2009a, 2009b). However, research has begun to extend the understanding of temporal self-perceptions beyond the continuity from the present to the future self (e.g., Rutt & Löckenhoff, 2016; Sokol & Eisenheim, 2016; Sokol & Serper, 2019c). The current review aimed to incorporate findings from three domains of temporal self-perceptions in emerging adults: (1) the perception of the future self, (2) changes in the perception of the future self across time, and (3) continuity of perceptions of multiple temporal selves (i.e., past–present–future). Specifically, this targeted review sought to highlight research exploring the longitudinal processes in these relationships (e.g., changes in temporal self-perceptions over time and the relationship between temporal self-perceptions and longitudinal outcomes).

## 2. Proposed Model

Since emerging adulthood was first introduced as a developmental stage, there has been discussion and contention in the literature about who the stage applies to (i.e., who has the opportunity to be an emerging adult as it was defined by Arnett (2000); see Arnett et al., 2011; Bynner, 2005; Cohen et al., 2003; Côté & Bynner, 2008; Hendry & Kloep, 2007; Syed & Mitchell, 2013). While social and economic changes in some societies have allowed for delays in adulthood, this may not be the case in other societies—or in subsets of societies (e.g., low income and racial/ethnic minorities)—where social norms and economic realities necessitate the transition from adolescence to adulthood. The vast majority of research on temporal self-perceptions and their implications during this developmental stage (i.e., ages 18 to 29) focuses on participants in North America (primarily the United States and Canada). As such, for the purposes of this review, we focus on emerging adulthood as it applies to individuals in industrialized societies. We return to this topic in the Conclusions Section to discuss limitations and future research directions.

A large portion of emerging adults in industrialized societies are college students, and it is relatively easy to study college students as a group. As such, much of the research on intertemporal decision-making in emerging adults focuses on college students. College students as a group commonly encounter intertemporal decisions (e.g., go out or study; persist in college or find a job and start earning money now), and the consequences of their decisions can be readily observed (e.g., receiving poor semester grades and changing their major/future goals). As such, college students present a good subsample to explore the relationships between perceptions of temporal selves and positive outcomes (e.g., psychological benefits and achievement) in emerging adults. This review highlights the findings that provide a foundation for understanding these relationships in college students and, when available, incorporates the additional literature that extends that understanding to emerging adults as a developmental group.

Importantly, college students are a heterogenous group that vary on many demographic factors (e.g., socioeconomic status and gender; Arnett & Tanner, 2006). For example, entering college, students vary in their socioeconomic backgrounds. A goal of higher education is to provide an opportunity to level the playing field across student backgrounds and support social mobility. For students from economically disadvantaged backgrounds, earning a college degree provides an avenue to move beyond their past difficulties (e.g., financial insecurity) and instead focus on their bright, positive future. Additionally, this heterogeneity extends to emerging adults as a developmental group. For example, within emerging adults, individuals vary widely in their educational path. Some pursue higher education at a university, some enroll in community college or trade school, while others enter the workforce. Given the heterogeneity of this developmental group, this review also incorporated findings that contribute to understanding the role of demographic factors in the relationships between perceptions of temporal selves and psychological and achievement outcomes. These findings may provide crucial information for efforts to support success in specific demographic groups.

**Figure 1:**
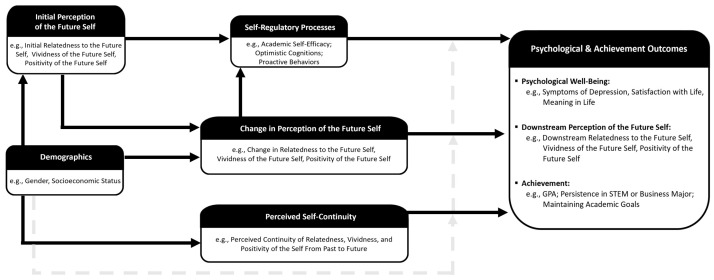
Proposed model of temporal self-perceptions and positive downstream consequences in emerging adults. Note: The solid arrows represent the relationships that are supported by the research reviewed below. The dashed arrow represents the potential for demographic factors to moderate the relationship between temporal self-perceptions and positive downstream outcomes. As discussed in detail below, the literature largely suggests that temporal self-perceptions are predictive of positive outcomes regardless of the demographic group. The exceptions to that general finding, as well as potential areas of future research addressing that relationship, are discussed below.

Therefore, the present review aimed to review evidence for a model demonstrating the connection between three domains of temporal self-perceptions —future self-perceptions, longitudinal changes in future self-perceptions, and continuity between temporal selves (i.e., past-to-future)—, self-regulatory processes, and positive downstream consequences (e.g., psychological well-being; academic success) in emerging adults. Figure 1 provides a model that incorporates the relationships between the three domains of temporal self-perceptions and crucial downstream psychological and achievement outcomes in emerging adults and the potential role of self-regulatory processes and demographic factors in those relationships. Below, we review the literature on each of the three temporal self-perception domains. We first provide an explanation of the construct and then highlight the evidence in the literature that demonstrates its relationship to positive outcomes in college students and in emerging adults in general, as proposed in the model. We then consider the role of self-regulatory processes as a mediational link in these relationships and demographic factors as a potential moderator. As we review the literature, we discuss the limitations and potential avenues for future directions in this growing area of research.

## 3. Temporal Self-Perceptions and Positive Outcomes

### 3.1. Perceptions of the Future Self

The literature on perceptions of the future self suggests that the construct is comprised of three distinct factors: (1) similarity and connectedness between the present and future self, (2) vividness of imagining the future self, and (3) positivity toward the future self (Bixter et al., 2020; Hershfield, 2011; Sokol & Serper, 2019a). Research on the factor structure of this construct demonstrated that each of these three factors contribute uniquely to the understanding of how an individual perceives their future self (Bixter et al., 2020; Sokol & Serper, 2019a). Additionally, research developing measures of perception of the future self has demonstrated its utility as a unique construct (Bixter et al., 2020; Sokol & Serper, 2019a). Although perception of the future self is correlated with important psychological factors such as self-control and self-esteem, research shows that perception of the future self predicts outcomes over and above these constructs. For example, ratings of positivity toward the future self predict future self-esteem after controlling for current self-esteem and relatedness to the future self predicts academic performance (i.e., GPA) even controlling for self-control (Bixter et al., 2020). These examples of divergent validity provide evidence that perceptions of the future self as a construct are more than simply a general measure of positivity toward the self or of general impulse control.

A main area of research on perceptions of the future self focuses on its ability to predict long-term outcomes. For example, in college students, research suggests that perceptions of the future self are important predictors of downstream academic performance (e.g., GPA), academic persistence (e.g., remaining in STEM and business majors), academic self-efficacy, interest in long-term financial well-being, depression symptoms, meaning in life, and satisfaction with life (see Bixter et al., 2020; McMichael et al., 2022, 2023, 2024; Sims et al., 2020; see Pawlak & Moustafa, 2023 for a review of the relationship between future thinking and academic outcomes). Research has also considered the role of perceptions of the future self in college students’ resilience during large-scale present challenges. Specifically, considering the COVID-19 crisis, the literature suggests that perception of the future self may be an important factor for maintaining both academic and career goals and mental health during a global crisis (McMichael & Kwan, 2023).

Focusing more broadly on emerging adults in general, the literature demonstrates that perceptions of the future self are predictive of reductions in intention to commit delinquent behaviors (e.g., cheating), self-reported delinquent behaviors (e.g., underage drinking), and procrastination (Blouin-Hudon & Pychyl, 2015; van Gelder et al., 2013, 2015). Additionally, perceptions of the future self are associated with increases in the likelihood of selecting a delayed reward, moral actions, psychological well-being, self-control, self-esteem, and mental health (Adelman et al., 2017; Bixter et al., 2020; Ersner-Hershfield et al., 2009a, 2009b; Klineberg, 1968; Simić et al., 2023; Sokol & Serper, 2019b).

Importantly, research has also demonstrated that experimentally manipulating perceptions of the future self leads to positive outcomes. For example, compared to control groups, after exposure to an aged avatar depicting their future self, college students were more likely to report stronger similarity to their future self, were more likely to allocate more financial resources to their future retirement, and indicated more interest in long-term financial well-being (Hershfield et al., 2011; Sims et al., 2020). Similarly, two studies manipulating vividness of the future self in emerging adults found that the experimental group was significantly less likely to report the intent to make a delinquent choice (e.g., intent to buy a potentially stolen laptop) and engage in cheating (van Gelder et al., 2013). Another study, with participants ranging from 16 to 19 years of age, found that interacting with a future self avatar over seven days led to increased vividness of the future self, which, in turn, predicted decreases in self-reported delinquent behaviors (e.g., stealing, drinking alcohol, and cheating; van Gelder et al., 2015). The malleability of future self-perceptions, in concert with its association with downstream outcomes, highlights that this is a potentially important construct for interventions to support emerging adults’ psychological well-being and achievement through both routine and large-scale challenges in their present lives.

### 3.2. Changes in Perceptions of the Future Self over Time

As demonstrated above, the literature clearly illustrates the positive outcomes associated with perceptions of the future self. Much of the literature focuses on the relationships between temporal self-perceptions and intertemporal decisions and longitudinal outcomes (e.g., saving money for the future, downstream mental health, academic performance, and delinquent behaviors; Bixter et al., 2020; Ersner-Hershfield et al., 2009a, 2009b; van Gelder et al., 2013). Despite the focus of this literature on longitudinal relationships and outcomes, until recently, few studies have examined the longitudinal changes in temporal self-perceptions. One study using a longitudinal design found that mean levels of vividness of the future self were relatively stable over the short term (i.e., two weeks; van Gelder et al., 2015). Similarly, an article developing a scale to measure perception of the future self reported a non-significant change in the mean levels of connectedness, vividness, and positivity toward the future self in college students over a five-week period (Bixter et al., 2020).

Given the clear relationship between temporal self-perceptions and the passage of time, recent research in this area has moved to explore longitudinal changes over longer periods, individual differences in those changes, as well as the relationships between those changes and critical outcomes. Using data from a longitudinal study of college students from their first week of college through their college career, a recent study suggests significant individual differences in changes in perception of the future self over time and points to the importance of these changes in predicting positive downstream outcomes (McMichael et al., 2022). Specifically, over the first two years of college, there were significant differences in how students changed in vividness of their college graduation and vividness of five years after their college graduation (i.e., the variances around the means were significant; McMichael et al., 2022). In both future domains, changes in vividness predicted academic self-efficacy, academic performance, and persistence in STEM and business majors. Similarly, in a subset of that longitudinal sample that included students graduating during the COVID-19 crisis, general vividness of the future increased significantly over the four years of college, and again, there were significant individual differences in that change (McMichael & Kwan, 2023).

These first steps into investigating longitudinal changes in perceptions of the future self in emerging adults suggest that not only are there changes over time, but those changes vary by individual and may be important for predicting long-term outcomes. Sources of those individual differences are a critical area for future research. One source of individual differences in change in perceptions of the future self may be demographic characteristics (e.g., gender and socioeconomic status). Below, we discuss in detail the role of demographics in these relationships and the literature supporting the existence of differences across demographic groups.

Importantly, in the existing literature, the models of change assume linear change in perceptions of the future self over time (i.e., perceptions either increase or decrease linearly). Perceptions of the future self may change non-linearly, and as such, the linear models may not sufficiently capture the degree and pattern of the change. Future research should explore this possibility and use alternative models to provide further clarity on the trajectory of the change. Understanding these relationships is crucial for clarifying the long-term functioning and benefits of temporal self-perceptions.

### 3.3. Temporal Self-Continuity

In recent history, perceptions of the future self have garnered extensive research attention, and the literature on that area of research continues to grow. However, another aspect of perceptions of temporal selves—temporal self-continuity—has long been a consideration of both philosophy and psychology (Erikson, 1963, 1968; Locke, 1690/2008). In fact, philosophy and psychology have traditionally proposed that a stable and consistent self is a critical factor for maintaining an individual’s psychological well-being and health (Erikson, 1968; Locke, 1690/2008). Given the extensive theoretical discussions surrounding temporal self-continuity, most empirical studies in this area focus on the degree of continuity between the present self and one temporal self (i.e., past-to-present and present-to-future; e.g., Burrow et al., 2020; Ersner-Hershfield et al., 2009a, 2009b).

More recently, research has moved to consider bi-directional continuity of temporal self-perceptions and its relationship to psychological outcomes (e.g., Rutt & Löckenhoff, 2016; Sokol & Eisenheim, 2016; Sokol & Serper, 2019c). These studies focus primarily on the perceived similarity between temporal selves—rather than vividness or positivity toward temporal selves— and have reported weak-to-moderate continuity (Sokol & Eisenheim, 2016). Studies that focused on past-to-present and present-to-future self-perceptions independently, suggested that both temporal directions may be important predictors of positive outcomes (e.g., Bartels & Rips, 2010; Bixter et al., 2020; Burrow et al., 2020; Ratner et al., 2020). However, a line of research focusing primarily on clinical populations that included both temporal directions at once suggested that similarity to the future self, but not the past self, was an important predictor of positive psychological outcomes (e.g., satisfaction with life and depression severity; Sokol & Serper, 2017, 2019b, 2019c).

Given the growing evidence connecting perceptions of the future self and positive long-term outcomes in emerging adults, an important direction for the literature is to test the relationship between this additional domain of temporal self-perceptions (i.e., continuity of temporal selves) and psychological and achievement outcomes. One recent line of research aimed to clarify those relationships by testing the degree of continuity in perceptions of the self from past-to-present-to-future in college students (McMichael et al., 2024). The findings suggest that while perceptions of the past self and perceptions of the future self were related, there was only weak-to-moderate continuity across time. Although the continuity of temporal self-perceptions was not strong, both past self- and future self-perceptions were important predictors of mental health and psychological well-being. In fact, both temporal self-perceptions uniquely contributed to predicting those psychological outcomes. Importantly, the results also pointed to differences in those relationships across demographic groups, which are discussed in detail below.

Overall, the results of this study built upon the understanding of continuity of temporal selves and highlighted the significance of multiple temporal selves to psychological outcomes (McMichael et al., 2024). Specifically, the results provided support for theoretical conceptions that perceived temporal self-continuity is an important factor in maintaining psychological well-being and health. Additionally, the finding that both past and future self-perceptions were significant and unique contributors in predicting positive psychological outcomes for college students enhances the previous understanding of the role of temporal self-continuity in psychological well-being. The majority of current findings in the literature focused on either college students or clinical samples. Future research should continue to explore these relationships in more diverse groups of emerging adults (e.g., individuals in the workforce and in trade schools).

### 3.4. Temporal Self-Perceptions and Self-Regulatory Processes

While much of the research on temporal self-perceptions focuses on measuring its relationship to long-term outcomes, researchers have also considered the process underlying those relationships. Specifically, several lines of research have identified self-regulatory processes as a potential mediator between perceptions of temporal selves and positive downstream outcomes. Here, we first briefly review the literature on the importance of self-regulatory processes and then discuss the literature in this field that provides support for the role of self-regulatory processes as a mediator in the proposed model (see Figure 1).

Self-regulatory processes encompass the mechanisms through which individuals manage their thoughts, feelings, and behaviors (Bandura, 1991). Self-efficacy— beliefs about one’s skills and abilities to succeed in a given goal or task—plays a central role in self-regulation processes (Bandura, 1977, 1981, 1991). Self-efficacy beliefs influence self-regulation processes by influencing individuals’ activity choices, motivation, effort expenditure, and persistence through difficulties.

Greater self-efficacy beliefs are associated with greater success in self-regulation. In contrast, lack of self-efficacy can predict decreases in motivation, avoidance of a task, and procrastination (Bandura, 1977, 1981, 1982, 1991; Wood & Bandura, 1989). For example, consider two students entering college for the first time. Both are highly motivated to graduate with their bachelor’s degree in computer science. One has high self-efficacy that they can achieve their goal, and the other does not. When presented with a choice to stay at home and study for an exam or go out to a party (i.e., an intertemporal decision), the student with greater belief in their ability to achieve their goal of attaining their degree is more likely to successfully self-regulate and make the decision to stay at home and study. Specific to emerging adults in college, greater self-efficacy predicts grade striving, effective time management, less academic disengagement, higher GPAs, and retention in college (Liem & Martin, 2012; Robbins et al., 2004; Schunk, 1984; Zimmerman et al., 1992). In a meta-analytic review of 109 studies, academic self-efficacy was the most important psychological predictor of GPA and college retention (Robbins et al., 2004).

The literature provides evidence for the relationship between temporal self-perceptions and self-regulatory processes (e.g., decreases in temporal discounting and increased self-control; Adelman et al., 2017; Bixter et al., 2020; Hershfield et al., 2011). Specific to emerging adults as a developmental group, there is a theoretical basis to expect a relationship between temporal self-perceptions and self-regulatory processes. In the literature, it is evident that specific and salient goals are important factors in the development of self-efficacy (Bandura & Schunk, 1981). Unfortunately, for individuals moving into a new developmental stage—such as emerging adults— goals often lack specificity and may be distal and vague. While goals may be for the distal future, the relationship between those goals and levels of self-efficacy may vary depending on the levels of perception of the future self. Emerging adults who perceive their future self vividly, positively, and as highly related to the present may see their future goals, even goals that are for the distal future, as clear and specific.

Recent research has begun to explore this hypothesis and expand upon the prior findings, suggesting a link between temporal self-perceptions and self-regulatory processes. Specifically, based on the longitudinal study following college students from the start of college described above, findings suggested that both initial levels of vividness of the future, and how that vividness changed over two years, predicted downstream academic self-efficacy (McMichael et al., 2022). This was true even after controlling for initial levels of academic self-efficacy, which lends support to a directional relationship with perceptions of the future self promoting greater self-efficacy. Additionally, in the subsample of the longitudinal study including only students graduating during COVID-19, greater initial future vividness predicted downstream proactive, self-regulatory behaviors (e.g., taking action to attain a job or graduate school acceptance prior to graduation) and, in turn, predicted persistence in academic and career goals despite sudden pressures in the present due to the spread of the global pandemic (McMichael & Kwan, 2023).

Taken together, the literature suggests a mediational link between temporal self-perceptions, self-regulatory processes, and positive downstream outcomes. Future research should continue to develop the understanding of this link in emerging adults in two avenues. First, research should expand the literature to test the relationship between aspects of temporal self-perceptions (e.g., future self-perception, change in future self-perception, and perceived self-continuity), self-regulatory processes, and a greater breadth of psychological and achievement outcomes (i.e., outcomes relevant to emerging adults on the whole rather than just college students). Second, future research should address the reciprocal effect between self-regulatory processes and future self-perception. In our model, we proposed that future self-perception affects academic self-efficacy. Nevertheless, self-efficacy could also affect future self-perception. Researchers should clarify the understanding of these relationships by employing experimental methods to test the directionality of the relationship between aspects of temporal self-perceptions and self-regulatory processes.

## 4. The Role of Demographic Factors

Despite belonging to the same developmental group, emerging adults in industrialized societies are far from homogenous. The diversity in their backgrounds and experiences may play a critical role in understanding their temporal self-perceptions. Lending initial support to this idea, in a study investigating perception of the future self and self-control in college students, researchers found that a crucial demographic factor—first-generation college student status—moderated the relationship between focus on the future and self-control (Adelman et al., 2017). There was a stronger positive relationship between future focus and self-control for college students who had a prior family member graduate from college (i.e., continuing-generation students) than those students who were first-generation students. Additionally, focusing on adults of all ages (from 18 to 73 years of age), a recent study found that socioeconomic status was predictive of the levels of future self-perception (Antonoplis & Chen, 2021). Specifically, individuals from higher socioeconomic backgrounds viewed their future more vividly, more positively, and more similar to the current self. This was true using both an observational method (i.e., the participants’ reported their socioeconomic status) and an experimental manipulation of socioeconomic status. Building on this foundation, recent research in this area has investigated potential differences in aspects of temporal self-perceptions by demographic group and sought to explore if the relationship between temporal self-perceptions and positive outcomes differed based on an individual’s background.

Specifically, to begin to understand the role of demographic backgrounds, recent lines of research have considered the relationship between demographic groups (e.g., gender and socioeconomic status) and the levels of future self-perceptions in emerging adults. In the longitudinal study described above, researchers tested if initial ratings of future self-perception as emerging adults began their college careers differed by gender or socioeconomic status. Regarding differences by gender, the results indicated that women may be at an advantage when entering college. In comparison to men, women began their college career reporting greater levels of vividness of both their college graduation and life five years after college graduation (McMichael et al., 2022). As noted above, these higher levels of initial vividness were predictors of academic self-efficacy, academic performance, and major persistence. Importantly, within the same longitudinal sample, vividness of the future at the start of college did not vary significantly by socioeconomic status (McMichael & Kwan, 2023). Students who were from a background characterized by economic need did not report lower vividness than students who were middle class or above. This finding differed from prior studies focusing on adults of all ages, which found that higher socioeconomic status did predict greater vividness of the future (Antonoplis & Chen, 2021). This difference in findings may result from the focus on college students rather than adults of all ages.

Beyond the relationship between demographics and initial levels of future self-perception, research has also considered the role of demographics in how perceptions of the future self changed over time. Again, the findings pointed to differences in change in vividness of the future over the first two years of college by gender (McMichael et al., 2022). Specifically, the positive change in women’s vividness of their lives five years in the future was significantly less than men’s. Recall that positive changes in future vividness were associated with positive academic outcomes, suggesting that men were at an advantage when considering these changes. Considering the relationship between socioeconomic status and changes in perceptions of the future self, research suggests that there were no significant differences in changes in vividness over the course of college by economic background (McMichael & Kwan, 2023). It is important to note that the non-significant differences in initial perceptions of the future self and in its change by socioeconomic status may be a result of relatively less power in this study. Future research should explore that relationship with greater power in order to provide greater clarity on how these relationships differ by specific demographic characteristics.

While the above findings suggested that perceptions of the future self may be stable across socioeconomic status, research has shown that economic background was a significant predictor of the continuity of temporal selves (i.e., the relationship between perceptions of the past self and perceptions of the future self; McMichael et al., 2024). For college students from high socioeconomic backgrounds, positive perception of the past self was an important predictor of positive future self-perception. This was not the case for students from lower socioeconomic backgrounds. These results suggest that for students with a background characterized by economic difficulty perceptions of their past self may be less crucial to their perception of a positive, successful future.

Finally, this area of research has begun to explore if the relationships between temporal self-perceptions and positive outcomes differed by demographic group. In other words, it investigates if the mechanism proposed in the model (see Figure 1) was the same regardless of gender or socioeconomic background. Taken together, the literature suggests few differences in the mechanism by gender or socioeconomic status. On the whole, a positive, similar, and vivid temporal self-perception was predictive of positive outcomes across groups. Specifically, the research found that greater vividness of the future, as well as greater increases in that vividness, predicted greater academic self-efficacy, academic performance, and STEM and business persistence for both men and women (McMichael et al., 2022). Similarly, regardless of socioeconomic status, greater vividness of the future at the start of college predicted proactive behaviors (e.g., attaining a graduate school or job acceptance) and persistence in academic and career goals during a global crisis (i.e., COVID-19; McMichael & Kwan, 2023). Additionally, perceptions of the past self and perceptions of the future self were jointly predictive of positive mental health and satisfaction with life across socioeconomic groups (McMichael et al., 2024).

Despite the overall similarity in the mechanism across demographic groups, there are three notable findings in the literature that may suggest potentially important differences in the mechanism by socioeconomic status: (1) Considering vividness of graduating college students before and during the pandemic, there was a significant difference by socioeconomic status in the relationship between vividness of the future at the start of college and the likelihood of having a job or graduate school acceptance at the start of the crisis. Specifically, for students from lower socioeconomic backgrounds, higher initial vividness significantly predicted having a job or graduate school acceptance at the start of the crisis. For higher socioeconomic status students, that relationship was still positive but not significant. This difference between socioeconomic groups was significant despite the relatively small sample sizes in this study, suggesting that vividness when entering college may be especially crucial for lower socioeconomic status students’ preparation for a crisis (McMichael & Kwan, 2023). (2) In the same study, considering the relationship between change in vividness of the future over college and vividness during COVID-19, there was a significant difference by socioeconomic status. For students from higher socioeconomic backgrounds, a greater positive change in vividness over the course of college significantly predicted having higher vividness at the start of the pandemic. This relationship was not significant for lower socioeconomic status students. This finding suggests that vividness during the crisis was supported by a positive change in vividness through their college career for high socioeconomic status students. For lower socioeconomic status students, who were likely encountering relatively more present challenges due to the pandemic, vividness during the global crisis was relatively independent of their prior changes in vividness. (3) Considering the predictive value of past and future self-perceptions, there was a significant difference in predicting meaning in life by socioeconomic status (McMichael et al., 2024). For higher socioeconomic status students, greater continuity in both temporal directions (i.e., past and future) predicted greater meaning in life. In contrast, for students from lower socioeconomic backgrounds, regardless of temporal direction, temporal self-perceptions did not predict ratings of meaning in life. This finding was not hypothesized in the research, but it suggests that lower socioeconomic status students may be less focused on the role of the self—past and future—when assessing their meaning in life. Importantly, this difference was not found when predicting depression symptoms or satisfaction with life, suggesting that there may be something unique about the relationship between temporal self-perceptions and meaning in life for lower socioeconomic status students.

Overall, these differences in the mechanism of temporal self-perceptions and positive outcomes by socioeconomic status were not hypothesized in the research. Developing a better understanding of these differences may be crucial for programs designed to support success for emerging adults from challenging economic backgrounds. Future research should seek to replicate these differences and further explore the relationships between socioeconomic status and temporal self-perceptions.

The demographic group research highlighted in this review focused on gender and socioeconomic status as these groups are often the focus of interventions intended to improve positive downstream academic outcomes (e.g., STEM persistence and college degree completion). The findings suggest differences in both initial levels of temporal self-perceptions and how those perceptions change by gender as well as differences in temporal self-continuity by socioeconomic status. Additionally, while the mechanism of temporal self-perceptions and positive outcomes held across gender, some mechanistic differences across socioeconomic status warrant additional research. Taken together, the findings highlight the importance of considering demographic background in temporal self-perception research and the need for additional research investigating these relationships across other demographic groups within emerging adults (e.g., ethnicity, college student versus workforce, and nationality).

## 5. Conclusions

The lines of research reviewed here considered the relationships between perceptions of temporal selves and positive outcomes in college students and emerging adults in general. It also explored the role of self-regulatory processes as a mediator in those relationships and demographic factors as a potential moderator. Taken together, the review provided support for the proposed model (see Figure 1), highlighting the importance of perceptions of temporal selves in predicting a range of positive long-term outcomes including self-regulatory processes (e.g., academic self-efficacy, optimistic cognitions, and proactive behaviors), psychological well-being (e.g., depression symptoms, meaning in life, and satisfaction with life), and achievement (e.g., cumulative grade point average, persistence in STEM and business majors, and maintaining academic goals). Additionally, the review demonstrated preliminary support for the significance of crucial demographic factors (e.g., gender and socioeconomic status) in understanding the nature of these relationships in emerging adults.

As highlighted throughout the review, as research on the relationship between perceptions of temporal selves and positive outcomes in emerging adults continues to grow, there are several clear avenues for future research. First, while research on longitudinal changes in future self-perceptions and continuity of temporal self-perceptions is gaining traction, the models in the current literature assume a linear pattern of change. While these models were a good starting point for understanding changes in temporal self-perceptions over time, temporal self-perceptions may be responsive to events occurring in the present leading to change patterns that are non-linear. For example, college students may start their college career with a positive and vivid view of their bright future. That perception of the future may suffer in the face of challenges occurring in the present (e.g., poor academic performance in their first semester). Students who are resilient to those present challenges (e.g., getting academic support and adjusting study habits) may see a subsequent increase in their future self-perceptions, while students who are unable to adjust may see continual decline. This example highlights present challenges that are routinely encountered by emerging adults. Other more large-scale present challenges (e.g., a global pandemic and economic decline) may lead to even greater changes in temporal self-perception trajectories for emerging adults. Given the potential for non-linear changes, the linear models in the current literature may not capture the full degree and pattern of changes. Future research in this area should continue to study longitudinal changes in temporal self-perceptions and aim to explore both linear and non-linear change trajectories. A clear understanding of those trajectories is key in understanding the downstream consequences of temporal self-perceptions.

Additionally, while the literature studying temporal self-perceptions and positive outcomes in emerging adults is growing, much of the current research focuses primarily on college students. Even within industrialized societies, emerging adults as a demographic group are diverse. For example, in terms of educational plans, some emerging adults are attending four-year universities, while some are pursuing community college or trade schools, and others join the workforce. As noted above, the vast majority of research on emerging adulthood and temporal self-perceptions focuses on emerging adults in industrialized societies—most often the United States and Canada. This limited geographic focus in the literature leads to questions about the generalizability of findings. For example, beyond industrialized societies, what it means to be a young adult (i.e., 18 to 29 years old) may differ (Bynner, 2005; Cohen et al., 2003; Côté & Bynner, 2008; Hendry & Kloep, 2007; Syed & Mitchell, 2013). Those differences may impact how the relationships in the proposed model function. Future research should continue to explore the relationships in the proposed model in more diverse groups of emerging adults both within industrialized societies and in societies where individuals may not have the social and economic support for exploration between adolescence and adulthood. Furthermore, while the literature currently demonstrates a clear relationship between aspects of temporal self-perceptions and psychological and achievement outcomes, many of the currently studied outcomes are most relevant to college students (e.g., GPA and changing major). Future research should expand those outcomes to include achievement outcomes that are relevant to other groups of emerging adults. Expanding the literature to include diverse groups of emerging adults and outcomes will be crucial to generalizing the findings in the literature across the demographic group.

Relatedly, the current literature has begun to consider the impact of demographic factors on the relationships between temporal self-perceptions and positive downstream outcomes. The findings in the literature emphasize that demographic background is a critical factor to consider in temporal self-perception research. Future research should consider both replicating findings in the literature (e.g., differences by socioeconomic status in the mechanism of temporal self-perceptions and positive outcomes) and expanding the literature by considering additional demographic groups within emerging adults (e.g., race/ethnicity, geographic location, culture, and educational path). Future research on the impact of race/ethnicity in the model may be particularly fruitful given (1) the cited evidence that demographic factors that are associated with race/ethnicity (e.g., socioeconomic status and college generations status) do have a relationship with temporal self-perceptions and how they relate to positive downstream outcomes and (2) the broader literature that indicates that social constructs such as race and colorism are related to self-perceptions (e.g., self-esteem and self-doubt; Moran, 2024; Sharif & Siddique, 2020; Sprecher et al., 2013). This type of future research will aid in understanding how demographic factors impact the mechanism underlying the relationship between temporal self-perceptions and positive outcomes and may inform programs that aim to support emerging adults in their long-term psychological well-being and achievement.

Finally, the longitudinal research in this field was a crucial first step in understanding how the domains of temporal self-perceptions relate to downstream positive outcomes in emerging adults. However, the current longitudinal research is largely observational, limiting the ability to make concrete statements about the directionality of the relationships. Future research should build upon this foundation by employing experimental methods within longitudinal research to clarify the mechanisms in these relationships.

## Data Availability

No new data were created or analyzed in this study. Data sharing is not applicable to this article.
